# An observational claims data analysis on the risk of maternal chronic kidney disease after preterm delivery and preeclampsia

**DOI:** 10.1038/s41598-021-92078-2

**Published:** 2021-06-15

**Authors:** Maren Goetz, Mitho Müller, Raphael Gutsfeld, Tjeerd Dijkstra, Kathrin Hassdenteufel, Sara Yvonne Brucker, Armin Bauer, Stefanie Joos, Miriam Giovanna Colombo, Sabine Hawighorst-Knapstein, Ariane Chaudhuri, Gudula Kirtschig, Frauke Saalmann, Stephanie Wallwiener

**Affiliations:** 1 Department of General Pediatrics, University Children’s Hospital, Heidelberg, Germany; 2 Department of Psychology, Ludwig Maximilian University, Munich, Germany; 3 Department of Women’s Health, University Hospital Tuebingen, Tuebingen, Germany; 4 Department of Obstetrics and Gynecology, University of Heidelberg, Heidelberg, Germany; 5 Institute for General Practice and Interprofessional Care, University Hospital Tuebingen, Tuebingen, Germany; 6 Department of Health Promotion, AOK Baden-Wuerttemberg, Stuttgart, Germany

**Keywords:** Kidney diseases, Health care, Epidemiology

## Abstract

Women with complications of pregnancy such as preeclampsia and preterm birth are at risk for adverse long-term outcomes, including an increased future risk of chronic kidney disease (CKD) and end-stage kidney disease (ESKD). This observational cohort study aimed to examine the risk of CKD after preterm delivery and preeclampsia in a large obstetric cohort in Germany, taking into account preexisting comorbidities, potential confounders, and the severity of CKD. Statutory claims data of the AOK Baden-Wuerttemberg were used to identify women with singleton live births between 2010 and 2017. Women with preexisting conditions including CKD, ESKD, and kidney replacement therapy (KRT) were excluded. Preterm delivery (< 37 gestational weeks) was the main exposure of interest; preeclampsia was investigated as secondary exposure. The main outcome was a newly recorded diagnosis of CKD in the claims database. Data were analyzed using Cox proportional hazard regression models. The time-dependent occurrence of CKD was analyzed for four strata, i.e., births with (i) neither an exposure of preterm delivery nor an exposure of preeclampsia, (ii) no exposure of preterm delivery but exposure of at least one preeclampsia, (iii) an exposure of at least one preterm delivery but no exposure of preeclampsia, or (iv) joint exposure of preterm delivery and preeclampsia. Risk stratification also included different CKD stages. Adjustments were made for confounding factors, such as maternal age, diabetes, obesity, and dyslipidemia. The cohort consisted of 193,152 women with 257,481 singleton live births. Mean observation time was 5.44 years. In total, there were 16,948 preterm deliveries (6.58%) and 14,448 births with at least one prior diagnosis of preeclampsia (5.61%). With a mean age of 30.51 years, 1,821 women developed any form of CKD. Compared to women with no risk exposure, women with a history of at least one preterm delivery (*HR* = 1.789) and women with a history of at least one preeclampsia (*HR* = 1.784) had an increased risk for any subsequent CKD. The highest risk for CKD was found for women with a joint exposure of preterm delivery and preeclampsia (*HR* = 5.227). These effects were the same in magnitude only for the outcome of mild to moderate CKD, but strongly increased for the outcome of severe CKD (HR = 11.90). Preterm delivery and preeclampsia were identified as independent risk factors for all CKD stages. A joint exposure or preterm birth and preeclampsia was associated with an excessive maternal risk burden for CKD in the first decade after pregnancy. Since consequent follow-up policies have not been defined yet, these results will help guide long-term surveillance for early detection and prevention of kidney disease, especially for women affected by both conditions.

## Introduction

Preterm birth is a major global health burden and leading cause of perinatal morbidity and mortality. Approximately 15 million babies are born preterm every year, with rates rising^1,2^. The majority of preterm deliveries occur spontaneously; approximately 30% are performed for medical reasons, including maternal preeclampsia or fetal growth restriction^3,4^. Preterm delivery not only poses a significant risk for adverse perinatal outcomes, it is also an important factor among pregnancy complications that predispose the mother to cardiovascular disease (CVD) and chronic hypertension later in life, analogous to preeclampsia^5–10^.

During the last few decades, there has been increasing evidence that women with a history of adverse pregnancy outcomes also have an elevated risk of developing a chronic kidney disease (CKD) or end-stage kidney disease (ESKD) later in life^11–18^. The prevalence of CKD is estimated at 10–15% of the world population and, as a major cause of morbidity, CKD is an important global health issue^19,20^.

Despite the clinical and public health significance, the pathomechanism underlying preterm delivery is not fully understood. Inflammation, infection, and complex immunologic abnormalities represent the main determinants that have been suggested^21–24^. When preterm birth is associated with placental malperfusion, mechanisms of vascular dysfunction come to the fore, with far-reaching consequences for the renal and cardiovascular system^25–27^. Two hypotheses have been proposed regarding the association between adverse pregnancy outcomes and the development of CKD and CVD: a pregnancy in itself, but especially if complicated by preeclampsia or preterm delivery, might be an early marker of underlying cardiovascular dysfunction in women with a corresponding risk profile; thereby, pregnancy serves as a “stress test” that exacerbates a preexisting pathologic condition^28,29^. Indeed, classic modifiable cardiometabolic risk factors such as hypertension, dyslipidemia, and diabetes are likely to play a role and evidently become manifest more often in women after a preterm delivery or preeclampsia^30–32^. On the other hand, adverse pregnancy outcomes might cause permanent vascular damage, leading to premature aging of the vascular system and thus to an increased risk of subsequent kidney disease and CVD^33^.

While studies have identified preterm delivery as a strong risk factor for later CVD, only few studies have addressed the association between preterm delivery and subsequent renal failure. Two Norwegian cohort studies demonstrated that having a preterm infant increased the relative risk of later ESKD in a healthy registry cohort^18,34^. Likewise, two retrospective cohort studies from Canada and Israel found significantly higher rates of ESKD hospitalization in women with either spontaneous or indicated preterm deliveries^35,36^. A recent Swedish nationwide cohort study demonstrated that women with a history of preterm delivery were at increased risk for subsequent CKD (aHR 1.39, CI 1.32–1.45) and ESKD (aHR 3.61, CI 2.03–6.39), regardless of preeclampsia or whether they delivered small-for-gestational-age children. The highest CKD risk rates were found for women with medically indicated deliveries, including preterm deliveries with preeclampsia (aHR 2.81, CI 2.46–3.20)^15,16^. Preeclampsia was itself recently identified as a strong risk factor for CKD and ESKD in large cohort studies^17,18,35,37^.

Despite increasing evidence for adverse renal outcomes, current research revealed that knowledge of long-term risks is relatively low among healthcare professionals; obstetricians tend to have a greater awareness of subsequent kidney disease after a complicated pregnancy, but they are not routinely involved in the long-term aftercare^38^. A study in Germany showed that, although the majority of obstetricians were aware of an increased renal risk after an unfavorable pregnancy outcome, their knowledge of follow-up care and counseling of these patients was insufficient^39^.

Using claims data from a statutory health insurance, the aim of our large-scale cohort study was to assess the risk of CKD in women with a history of preterm delivery and to determine how the risk differs depending on CKD severity or when preeclampsia is present concurrently.

## Methods

### Study design and population

Our analyses were part of a large retrospective observational database study. We obtained a dataset with claims data from the AOK Baden-Wuerttemberg. The AOK Baden-Wuerttemberg is one of the major regional German statutory health insurance companies, covering 4.5 million insured persons. Women with singleton live births between January 1, 2010, and December 31, 2017, were identified. Regarding birth outcome, we analyzed a sample of mothers whose data could be matched with the data of their child/children (222,779 women with 291,091 births). Claims data from the AOK registry were also used to identify women who developed CKD or ESKD during follow-up, until September 30, 2019. Relevant billing coding both in primary care and in the outpatient sector was used to identify exposure and outcome variables. Claims data sets contained outpatient data from 2007, the full dataset including inpatient data was available from 2008 onwards. All diagnoses were encoded by the International Classification of Diseases (ICD-10 German Modification). Additionally, OPS (Operation and Procedure Classification System) and DRG (Diagnosis Related Groups) codes were used. A full list of ICD, OPS, and DRG codes is presented in Table S1 (Appendix).

We considered the births as the statistical subject for the current analyses. Due to miscoding of the quarter of birth in the claims data, we had to exclude pregnancies with implausible delivery times (*n* = 964) from all analyses. We also excluded births of multiples (*n* = 5,872) since they are more likely to be delivered preterm than are singleton pregnancies. Moreover, births with implausible records for maternal age (*n* = 23) were excluded, as were patients who were not insured with the AOK for < 40% of the observation period (*n* = 26,365). To avoid further potential confounding, births with preexisting maternal diagnoses of CKD, ESKD, or KRT (between January 1, 2007, and September 30, 2019) were excluded at baseline (*n* = 863). Overall, 33,610 births were excluded (in some births more than one exclusion criterion applied) and the final sample consisted of 193,152 women with 257,481 births.

This study is reported as per the Strengthening the Reporting of Observational Studies in Epidemiology (STROBE) guideline (see Table S2, Appendix). Patient identification numbers that were originally used for linking files within the insurance databases were encrypted; all data were anonymized and nonidentifiable to the researchers. Ethical approval was obtained from the Ethics Committee of Tuebingen University Medical Faculty and University Hospital.

### Study variables

#### Exposure variables

The main exposure of interest was a history of preterm delivery, defined as any delivery with a gestational age of < 37 weeks. Gestational age was either determined based on the last menstrual period or by measuring the crown-rump length by transvaginal ultrasound. Preterm deliveries were categorized as “extremely preterm” for gestational age of < 28 weeks, “very preterm” for gestational age of 28 to < 32 weeks, and “moderate to late preterm” for gestational age of 32 to < 37 weeks. According to ICD coding for disorders associated with short gestation and low birth weight, gestational age at birth cannot be distinguished in detail, but only between < 28 weeks and ≥ 28 to < 37 weeks of gestation. Due to only a small number of cases in the former group, all preterm deliveries were combined into one single exposure variable “preterm delivery”.

We considered preterm delivery as a time-dependent variable so that a woman could contribute pregnancies and person-time to both unexposed and exposed groups during follow-up. For the main analysis, births were stratified into term birth vs. preterm delivery at gestational age < 37 weeks. Women were considered unexposed (i) if they had never delivered preterm, or (ii) had not delivered preterm from the date of their first term birth until the date of their first preterm delivery, irrespective of subsequent pregnancy outcomes. For example, if a woman had two deliveries during the study period and only her second delivery was preterm, she was considered unexposed between her first and second delivery, but from then on was considered exposed.

The second time-dependent exposure of interest was preeclampsia. Preeclampsia was defined as new onset of hypertension ≥ 140/90 mmHg after 20 weeks of gestation with at least one significant end-organ dysfunction, typically kidney involvement with proteinuria^40^. On the basis of ICD coding, it was impossible to classify preeclampsia as mild or severe disease. Additionally, we were not able to determine whether a preterm delivery was a spontaneous or medically indicated delivery. Hence, we determined preterm birth with concurrent preeclampsia as a surrogate parameter for a severe form of a medically indicated preterm birth.

Thus, we finally stratified the births into four groups according to the status of the two binary prior risk exposures: (i) neither any exposure to preterm delivery nor any exposure to preeclampsia, (ii) no exposure to preterm delivery but exposure to at least one preeclampsia, (iii) exposure to at least one preterm delivery, but no exposure to preeclampsia, or (iv) joint exposure to preterm delivery and preeclampsia.

### Outcome variables

CKD was the main outcome of interest. CKD was defined as a diagnosis of CKD, ESKD, or initiation of dialysis recorded in the health insurance database during follow-up. We categorized CKD according to disease severity. “General CKD” comprised the total quantity of cases with any CKD, including all CKD stages and ESKD cases, and also unspecified renal insufficiency. “Mild to moderate CKD” was defined as CKD stages 1–3, “Severe CKD” included CKD stage 4 and stage 5 (ESKD), initiation of dialysis, and (status after) kidney transplantation. The ICD and OPS codes used for each category are shown in Table 1.

**Table 1:** Outcome variables.

Category	ICD codes	OPS codes
“mild to moderate CKD”	N18.1, N18.2, N18.3, N18.81, N18.82, N18.83	
“severe CKD”	N18.0, N18.4, N18.5, N18.84, Z49.1, Z49.2, Z94.0, Z99.2	5-555, 8-853, 8-854, 8-855, 8-857
“general CKD”	N19, N18.8, N18.80, N18.9, N18.1, N18.2, N18.3, N18.81, N18.82, N18.83, N18.0, N18.4, N18.5, N18.84, Z49.1, Z49.2, Z94.0, Z99.2	5-555, 8-853, 8-854, 8-855, 8-857

Change of ICD code N18.- in 2010; both versions of ICD coding for CKD (before and after 2010) were used for the analyses.

### Covariates

We adjusted for the following confounding covariates: maternal age, diabetes, or gestational diabetes as well as obesity and dyslipidemia. Maternal exposure to diabetes (type 1 and 2) or gestational diabetes was determined by such a diagnosis having been recorded in the claims database. To identify obese women, we used ICD diagnoses of adiposity, overnutrition, and dyslipidemia as recorded. Adiposity was classified according to WHO grades. These preexisting conditions were time-dependent variables, where women were considered exposed from the date of their first delivery with a diagnosis of diabetes, adiposity, or dyslipidemia, respectively.

### Statistical analysis

The statistical analyses were performed using R version 4.0.2 and R-Studio v. 1.3.1056 for Windows (32/64 bit)^41^. The date of the first delivery marked the entry in the observation period for each woman. The study cohort was followed up for the earliest diagnosis of CKD from delivery to either death, migration, or end of the study period. Thus, using these survival data (i.e., data regarding the occurrence of time-dependent events and censored data) we aimed to compare risk groups (at least one risk exposure such as preterm birth and/or preeclampsia) with a reference group (no risk exposures), adjusted for defined binary and parametric covariates.

Cox proportional hazard regression was used to compute hazard ratios and 95% confidence intervals. In our proportional hazard model, the risk groups (ii – iv) were compared to the reference group of individuals who were not exposed to preterm delivery or to preeclampsia (i). The *HR*s were interpreted like relative risks: In our design a *HR* = 1 meant that there was no difference in the event rate between a risk group and the reference group free of risk. A *HR* > 1 meant that the event rate of a risk group was > 1 times the event-rate of the reference group without risk. A *HR* < 1 meant that the event rate of a risk group was < 1 times the event-rate of the reference group without risk. The model was adjusted for confounders, such as maternal age, diabetes or obesity, and dyslipidemia. We set the critical α-error to α ≤ 0.01. However, as the sample size was extensive, and thus the statistical power to detect even small effects (*HR*s ≈ 1) amounted to nearly 1-β = 1.00, we cannot conclude that any substantial meaning could be attributed to an effect just because of its statistical significance. Thus, in this study, we focused on the interpretation of the effect sizes, i.e., the *HR*s. In the final study population, there were no missing values regarding the variables used in the models (see Table 2).

**Table 2:** Sample characteristics.

		*f*	%
Number of births	1	134,805	52.36
	2	101,625	39.47
	≥ 3	21,051	8.18
	Missing	0	0.00
Birth mode	C-Section	80,635	31.32
	Vaginal	175,679	68.23
	Undefined	85	0.03
	Missing	1,082	0.42
Obesity / dyslipidemia	False	206,541	80.22
	True	50,940	19.78
	Missing	0	0.00
(Gestational) Diabetes	False	211,730	82.23
	True	45,751	17.77
	Missing	0	0.00
Preterms	False	240,533	93.42
	True	16,948	6.58
	Missing	0	0.00
Preeclampsia	False	243,033	94.39
	True	14,448	5.61
	Missing	0	0.00
Mild / moderate CKD	False	255,904	99.39
	True	1,577	0.61
	Missing	0	0.00
Severe CKD	False	257,291	99.93
	True	190	0.07
	Missing	0	0.00

*f* = frequency; % = percentage of *N* = 257,481.

### Ethics approval and consent to participate

Ethical approval was obtained from the Ethics Committee of Tuebingen University Medical Faculty and University Hospital. The Ethics Committee of Tuebingen University granted an exemption from requiring informed consent and confirmed that there were no objections to the analysis of sufficiently anonymized aggregated data sets.

## Results

### Sample characteristics

The study population consisted of 193,152 women with 257,481 singleton live births. Mean maternal age at delivery was 30.68 years (*SD* 5.27 years). The average interpregnancy intervals amounted to 2.76 years (*SD* 1.25 years). Mean observation time was 5.44 years. Further characteristics of the study population are listed in Table 2.

There were 16,948 preterm deliveries (6.58%) and 14,448 pregnancies (5.61%) with a diagnosis of preeclampsia. In all, 1,821 women (0.71%) were diagnosed with any CKD, of whom 1,577 were diagnosed with a CKD stage 1–3 (0.61%); 190 (0.07%) women developed a severe CKD, including ESKD. Mean age at any CKD diagnosis was 30.51 years (*SD* = 5.60 years) and did not differ according to CKD severity (CKD stages 1–3: *M* = 30.51 years, *SD* = 5.60 years; CKD stages 4–5 and ESKD: *M* = 30.51 years, *SD* = 5.60 years).

Regarding our four strata according to the status of the two binary prior risk exposures, there were:

i. 228,214 with neither an exposure of preterm delivery nor an exposure of preeclampsia,
ii. 12,319 with no exposure of preterm delivery but exposure of at least one preeclampsia,
iii. 14,819 with an exposure of at least one preterm delivery but no exposure of preeclampsia, and
iv. 2,129 with a joint exposure of preterm delivery and preeclampsia.

### Main analysis

**Figure 1:**
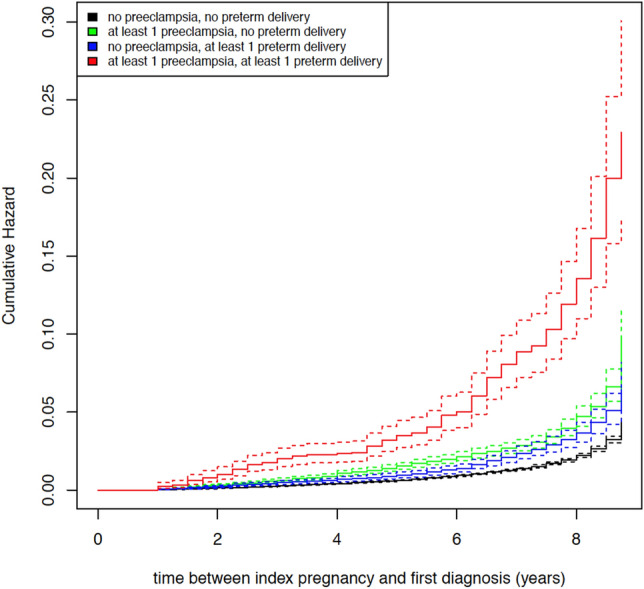
Cumulative hazard plot on risk of any subsequent CKD.

Compared to women with no risk exposure, the CKD risk was significantly increased for women, who had ever given birth affected by preterm delivery (HR 1.789, 95% CI [1.531; 2.091]). Women with a history of preeclampsia occurring at any time during pregnancy also had an increased risk of a subsequent CKD (HR 1.784, 95% CI [1.516; 2.098]), irrespective of CKD severity (see Table 3). The highest increase in risk was observed for women with a combined exposure to preeclampsia and preterm delivery: these women had a more than fivefold risk for subsequent CKD (HR 5.227, 95% CI [4.201; 6.504]) compared to women without any risk exposure. The effects of diabetes and obesity were comparable to the effects of the single risk exposures (diabetes *HR* = 1.470, obesity *HR* = 1.609; see Table 3). Only the effect of maternal age within the observation period seemed negligible (*HR* = 1.051). The cumulative hazard plot regarding the risk of any subsequent CKD is depicted in Fig. 1.

**Table 3:** Cox regressions for CKD by risk exposures.

Outcome	Exposure	*HR*	95% *CI HR*	*z*	*P*( >|*z*|)
Any CKD^1,2^	Preeclampsia alone^5^	1.784	[1.516; 2.098]	6.981	< .001
	Preterm delivery alone^5^	1.789	[1.531; 2.091]	7.316	< .001
	Both/joint risk exposure(s)^5^	5.227	[4.201; 6.504]	14.828	< .001
	Maternal age	1.051	[1.042; 1.060]	11.619	< .001
	Diabetes	1.470	[1.320; 1.638]	7.014	< .001
	Obesity	1.609	[1.453; 1.781]	9.138	< .001
Mild to moderate CKD^1,3^	Preeclampsia alone^5^	1.753	[1.471; 2.089]	6.268	< .001
	Preterm delivery alone^5^	1.806	[1.529; 2.133]	6.956	< .001
	Both risk exposures^5^	5.138	[4.060; 6.502]	13.626	< .001
	Maternal age	1.056	[ 1.047; 1.066]	11.874	< .001
	Diabetes	1.483	[1.322; 1.665]	6.698	< .001
	Obesity	1.635	[1.465; 1.823]	8.817	< .001
Severe CKD^1,4^	Preeclampsia alone^5^	2.971	[1.874; 4.711]	4.630	< .001
	Preterm delivery alone^5^	4.705	[3.259; 6.794]	8.263	< .001
	Both risk exposures^5^	11.903	[6.902; 20.528]	8.907	< .001
	Maternal age	1.021	[0.995; 1.048]	1.572	0.116
	Diabetes	1.181	[0.832; 1.674]	0.931	0.352
	Obesity	1.479	[1.074; 2.035]	2.400	0.016

*z* = *z*-Value; *P*( >|*z*|) = empirical significance level.

*CKD* Chronic kidney disease, *HR* Hazard ratio, 95% *CI HR* 95% confidence interval of hazard ratio.

^1^*N* = 257,481.

^2^Number of events = 1.821.

^3^Number of events = 1.577.

^4^Number of events = 190.

^5^Reference group = no exposure either to preterm delivery or to preeclampsia.

**Figure 2:**
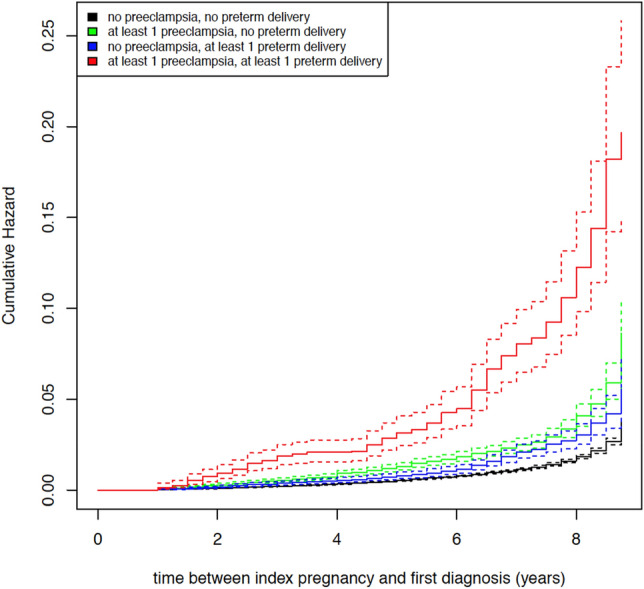
Cumulative hazard plot on risk of mild to moderate subsequent CKD.

**Figure 3:**
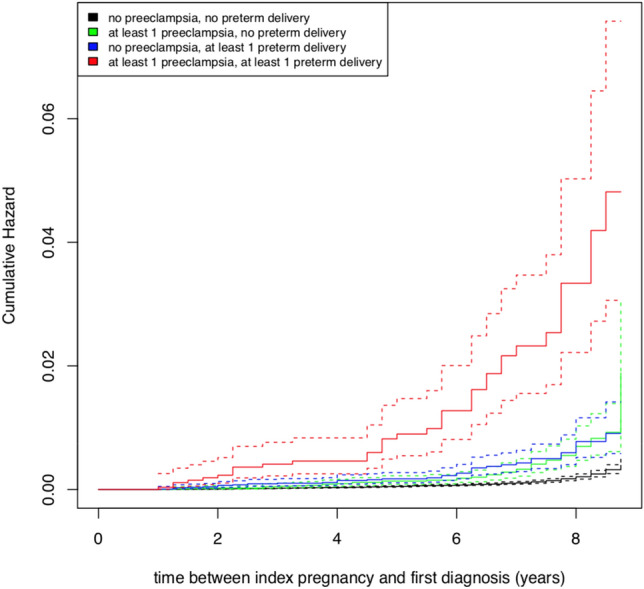
Cumulative hazard plot on risk of severe subsequent CKD.

These effects were the same in magnitude only for the outcome of mild to moderate CKD (see Table 3). However, the estimated *HR*s for the outcome of severe CKD exceeded the respective effects from prior models: Compared to women with no risk exposure, women with a history of at least one preeclampsia alone had an almost threefold risk (HR 2.971, 95% [1.874; 4.711]) for subsequent severe CKD compared to women without any risk exposure (see Table 3). For a history of preterm delivery, the risk was significantly increased (HR 4.705, 95% CI [3.259; 6.794]), with an almost fivefold higher risk compared to women without any risk exposure. Again, women with a combined exposure had the highest risk: Their chance of subsequently developing severe CKD was almost 12 times that of women without any exposure. As shown in Table 3, the *CI*s of the effects were wider in this model than in the latter two. Maternal age, diabetes, and obesity even failed to reach statistical significance in this model. Figures 2 and 3 depict the cumulative hazard plots regarding the risk of mild to moderate and severe subsequent CKD.

## Discussion

### Principal findings

Our analyses aimed to determine whether preterm birth poses a risk for subsequent CKD, either as a single risk factor or jointly with preeclampsia. Our results revealed that women with a history of preterm delivery had an increased risk for later CKD compared to women with term deliveries. A particularly strong association was found for a history of preterm delivery and subsequent, severe CKD, including ESKD, with a 4.7-fold higher risk. Considering births complicated by preeclampsia, there was also an increased risk for severe CKD and ESKD, but the association was less marked than for preterm births. Women who experienced a preterm delivery concurring with preeclampsia were at the highest risk for developing any CKD with a *HR* of 5.2. The risk of developing a severe CKD or ESKD after a preterm birth complicated by preeclampsia was even 11-fold higher.

### Comparison with previous research

The associations demonstrated are consistent with previous cohort studies which found an increased risk for renal insufficiency after a preterm delivery. A recent meta-analysis suggested that preterm delivery may be independently associated with a higher risk of maternal ESKD. Preterm preeclampsia was associated with the highest risk rates for ESKD (aRR, 5.66), and the association with ESKD persisted for women who had preterm deliveries without preeclampsia (aRR, 2.09)^16^. Consistently, we found the strongest association for later severe CKD in women with a history of preterm delivery with preeclampsia, with a 11.9-fold increased risk.

Although similar studies have been conducted, the risk rates are not directly comparable. Previous studies failed to adjust for important confounders or explicitly included only women with preexisting conditions. In addition, existing research mainly focused on ESKD incidence and ESKD hospitalization^18,34–36^. A recent cohort study by Barrett et al. was the first to report an association between preterm delivery and maternal CKD. They found the highest risk of CKD in women who experienced preterm delivery and preeclampsia (aHR 2.81, CI 2.46–3.20). However, spontaneous preterm delivery as a single exposure was also associated with an increased risk of CKD (aHR 1.32, CI 1.25–0.139), independent of preeclampsia or having delivered a small-for-gestational-age baby^15^. This population-based study from Sweden included 1,943,716 women, as compared to our cohort of 257,481 births. Due to database limitations, we were not able to stratify risk according to gestational age, but we also distinguished preterm deliveries as spontaneous or iatrogenic preterm, which in our case meant a preterm birth complicated by preeclampsia. Our analysis could corroborate previous results and could likewise identify preterm delivery as an important obstetric risk factor for subsequent CKD. As in our study, Barrett et al. also found the highest risk for joint exposure to preterm birth and preeclampsia, regardless of CKD severity^15,17^. Preeclampsia itself has recently been identified as a strong risk factor for maternal CKD and ESKD^14,17,37,42^. Our analysis revealed comparable risk rates for preeclampsia or preterm delivery as single risk exposure for any CKD.

With a study period of 10 years and a mean observation time of 5.44 years, the observation period in our study was comparably short. Mean follow-up of previous cohort studies was 7–27 years^15,17,18,43^. Despite our shorter time span, we were able to demonstrate clear trends for subsequent maternal CKD. Our results showed that after a complicated pregnancy, the first decade plays a particularly important role in the trajectory of renal failure. Kristensen et al. demonstrated a similar tendency in a nationwide cohort study on the risk of later kidney disease after a history of preeclampsia. They found that preeclampsia was especially strongly associated with the risk of CKD within five years of the latest pregnancy; thereafter, the strength of the association decreased^43^.

### Interpretation

The underlying cause of preterm birth is uncertain; often a “proinflammatory phenotype” is assumed^27^. Similarly, all factors involved in the metabolic syndrome are associated with low-grade inflammation^44^. Metabolic risk factors such as hypertension, obesity, and insulin resistance have been shown to be associated with an increased risk of preterm delivery and preeclampsia. Placental dysfunction is a key feature of both disease patterns^31,45–48^. It enhances the release of proinflammatory and antiangiogenic factors in the body^33^. This could explain the significantly increased risk we found in women with preeclamptic preterm deliveries. Proinflammatory processes contribute to accelerated endothelial dysfunction and systemic atherosclerosis, culminating in impaired end-organ function. In addition to the cardiovascular system, the renal system is particularly affected. Considering the shared underlying pathomechanisms, it seems plausible that preterm birth is a manifestation of a subclinical predisposition to later kidney disease in women with a corresponding risk profile^15^. However, our findings of severe CKD in women with a history of spontaneous preterm deliveries in the first decade after pregnancy suggest that preterm delivery may also be associated with early kidney damage^48^.

### Strengths and limitations

This study is the first to investigate maternal CKD after a preterm delivery in a German cohort. The key strength is the large cohort: over 190,000 women of childbearing age were followed up for almost a decade. The cohort size provided adequate power to examine the impact of preterm delivery independently of recognized risk factors. The prevalence of CKD was low (0.07–0.61%), which may be due to the fact that our cohort was comparably young for a disease that aggravates gradually over decades. Moreover, we cannot rule out the possibility that cases of early, subclinical CKD might not have been detected.

Regarding the generalizability of our results, we found similar prevalence rates of preterm births and cesarean sections as in national comparisons^49,50^. However, the study population consisted primarily of white European women living in Southern Germany, which limits the generalizability of our results for other ethnic groups.

To avoid possible confounding, women with pre-pregnancy kidney disease, renal insufficiency, or KRT were excluded from the analyses. Cases of pre-existing CVD or autoimmune diseases without any form of kidney involvement were not excluded, which may have confounded our results; we assume, however, that pre-pregnancy CVD rates were negligible in a cohort of young women of childbearing age, who tend to be healthy. We were also able to adjust for certain metabolic risk factors, such as obesity, diabetes, and dyslipidemia, which have all been associated with both adverse pregnancy outcomes and CKD^16,44^. However, no data for the exact BMI were available in the claims data registry. It can be assumed, however, that the coding for obesity was conservative since it has little billing relevance. Additionally, we did not have data on smoking, which is a risk factor for preterm delivery and CKD; however, studies have shown an inverse association between cigarette smoking during pregnancy and incidence of preeclampsia^51–53^. Therefore, we assume that the CKD risk increase cannot be attributed to the smoking status.

One limitation which applies to most registry-based studies is the questionable validity of recorded diagnoses. Since diagnoses were ascertained by medical professionals and not self-reported, we assumed correct coding in both primary care and specialist settings. However, incomplete or incorrect coding cannot be ruled out, especially for diagnoses with little billing relevance. In addition, claims data based on ICD-10 coding are limited in their ability to assess disease severity. To mitigate this effect, we used surrogate parameters for severity level (e.g., joint exposure of preeclampsia and preterm delivery as a surrogate for a severe form of a medically induced preterm birth).

Since we had no information about parity, we used the first pregnancy during the study period as the index pregnancy. This might have led to misclassification for the proportion of women who gave birth prior to our analysis window.

Regarding the analyses on severe forms of CKD, with a total number of just *n* = 190 events, the standard errors of the *HR*s was higher than the analyses regarding general or mild to moderate forms of CKD, making our estimations less exact in that specific model.

In general, the association between preterm delivery and maternal CKD must be considered correlative and not causal because, despite adjustment, we cannot completely rule out the influence of other important confounding factors.

### Implications

Despite the highly increased risk rates shown, the absolute risk of developing a CKD or ESKD after a preterm delivery remains low overall. However, the relative increase in risk among women who have been exposed to preterm delivery is clearly relevant from a population-based perspective, as preterm deliveries still account for a significant proportion of all births. Although an increasing number of professional societies recognize that an adverse pregnancy outcome strongly contributes to women’s overall risk profile for future chronic diseases, there is still a lack of structured follow-up. International guidelines show excessive variation in recommendations for long-term surveillance and screening eligibility^54^. Existing prediction models for CKD and ESKD do not consider obstetric history, although it is easy to assess in the clinical setting^55^. Identifying certain adverse pregnancy outcomes, particularly preterm delivery, as risk markers for future maternal CKD could provide an opportunity for early preventive measures for women at risk for CKD at a time when it might still be possible to alter the patient’s risk trajectory^11,56^.

## Conclusions

Any history of preterm delivery is associated with an increased risk for subsequent maternal CKD. Women with a history of preterm delivery and preeclampsia as joint exposure represent the main risk group for developing a CKD. So far, regular maternity care does not address this relevant problem adequately. Prevention strategies must be developed and established to reduce adverse long-term outcomes in affected women. Further research investigating the effectiveness and optimal timing of engaging women in systematic renal follow-up and including renal follow-up in existing primary care prevention programs is needed.

## Supplementary Information

Supplementary Information 1.

Supplementary Information 2.

Supplementary Information 3.

## Data Availability

The data that support the findings of this study are available from AOK Baden-Wuerttemberg but restrictions apply to the availability of these data, which were used under license for the current study, and so are not publicly available. Data are however available from the authors upon reasonable request and with permission of AOK Baden-Wuerttemberg.

## Abbreviations

AOK: Allgemeine Ortskrankenkasse (general local health insurance)
CKD: Chronic kidney disease
CVD: Cardiovascular disease
DRG: Diagnosis related groups
ESKD: End-stage kidney disease
ICD: International classification of diseases
KRT: Kidney replacement therapy
OPS: Operation and procedure classification system
