# Supplementary material for: An observational claims data analysis on the risk of maternal chronic kidney disease after preterm delivery and preeclampsia
Source: Sci Rep. 2021 Jun 15;11:12596. doi: 10.1038/s41598-021-92078-2 (PMC8206322; doi:10.1038/s41598-021-92078-2)
Supplement: Supplementary file 1 — Supplementary Information 1. [file 41598_2021_92078_MOESM1_ESM.docx]

Supplementary Table S1. List of codes for disease definitions.

|  | **ICD-10 codes** | **DRG codes** | **OPS codes** |
| --- | --- | --- | --- |
| Delivery outcome | O36.4, O60.1, O60.2, O60.3, O75.7, O80, O81, O82, Z37.-, Z38.- | O01, O02, O60 | none |
| Preeclampsia (exposure) | O14.- | none | none |
| Preterm delivery (exposure) | P07.- | none | none |
| Any pre-existing CKD, ESKD or kidney replacement therapy (exclusion) | N17.-, N18.-, N18.80, N18.81, N18.82, N18.83, N18.84, N19, Z49.1, Z49.2, Z94.0, Z99.2 | none | 5-555.x, 8-853.x, 8-854.x, 8-855.x, 8-857.x |
| “mild to moderate CKD” (outcome) | N18.1, N18.2, N18.3, N18.81, N18.82, N18.83 | none | none |
| “severe CKD”, including ESKD (outcome) | N18.0, N18.4, N18.5, N18.84, Z49.1, Z49.2, Z94.0, Z99.2 | none | 5-555.x, 8-853.x, 8-854.x, 8-855.x, 8-857.x |
| Adiposity, overnutrition | E66.-, E67.8, E68 | none | none |
| Dyslipidemia | E78.- | none | none |
| Diabetes | E10.-, E11.-, E12.-, E13.-, E14.-, | none | none |
| Gestational diabetes | O24 | none | none |
| low birthweight | P05. | P03, P04, P05, P61, P62, P63Z, P64Z, P55, P66 | none |
