# Supplementary material for: An observational claims data analysis on the risk of maternal chronic kidney disease after preterm delivery and preeclampsia
Source: Sci Rep. 2021 Jun 15;11:12596. doi: 10.1038/s41598-021-92078-2 (PMC8206322; doi:10.1038/s41598-021-92078-2)
Supplement: Supplementary file 3 — Supplementary Information 3. [file 41598_2021_92078_MOESM3_ESM.docx]

**An observational claims data analysis on the risk of maternal chronic kidney disease after preterm delivery and preeclampsia**

Maren Goetz^1^, Mitho Müller^2^, Raphael Gutsfeld^2^, Tjeerd Dijkstra^3^, Kathrin Hassdenteufel^4^, Sara Yvonne Brucker^3^, Armin Bauer^3^, Stefanie Joos^5^, Miriam Giovanna Colombo^5^, Sabine Hawighorst-Knapstein^6^, Ariane Chaudhuri^6^, Gudula Kirtschig^6^, Frauke Saalmann^6^, Stephanie Wallwiener^4*^

^1^Department of General Pediatrics, University Children's Hospital, Heidelberg, Germany

^2^Department of Psychology, Ludwig Maximilian University, Munich, Germany

^3^Department of Women’s Health, University Hospital Tuebingen, Germany

^4^Department of Obstetrics and Gynecology, University of Heidelberg, Heidelberg, Germany

^5^Institute for General Practice and Interprofessional Care, University Hospital Tuebingen, Germany

^6^Department of Health Promotion, AOK Baden-Wuerttemberg, Stuttgart, Germany

***Corresponding author:**

Stephanie Wallwiener, PhD,

Department of Obstetrics and Gynecology, University of Heidelberg, Im Neuenheimer Feld 440 Heidelberg, 69120 Germany,

Phone: 49 6221 5636956, Email: stephanie.wallwiener@med.uni-heidelberg.de
